# Measurement properties of depression questionnaires in patients with diabetes: a systematic review

**DOI:** 10.1007/s11136-018-1782-y

**Published:** 2018-02-02

**Authors:** Susan E. M. van Dijk, Marcel C. Adriaanse, Lennart van der Zwaan, Judith E. Bosmans, Harm W. J. van Marwijk, Maurits W. van Tulder, Caroline B. Terwee

**Affiliations:** 1 Department of Health Sciences, Faculty of Earth and Life Sciences, Amsterdam Public Health Research Institute, VU University Amsterdam, Amsterdam, The Netherlands; 2 Department of General Practice and Elderly Medicine and the Amsterdam Public Health Research Institute, VU University Medical Center, Amsterdam, The Netherlands; 3 Manchester Academic Health Sciences Centre and NIHR School for Primary Care Research, The University of Manchester, Manchester, UK; 4 Department of Epidemiology and Biostatistics and the Amsterdam Public Health Research Institute, VU University Medical Center, Amsterdam, The Netherlands

**Keywords:** Diabetes, Depression questionnaires, Measurement properties, COSMIN checklist

## Abstract

**Purpose:**

To conduct a systematic review on measurement properties of questionnaires measuring depressive symptoms in adult patients with type 1 or type 2 diabetes.

**Methods:**

A systematic review of the literature in MEDLINE, EMbase and PsycINFO was performed. Full text, original articles, published in any language up to October 2016 were included. Eligibility for inclusion was independently assessed by three reviewers who worked in pairs. Methodological quality of the studies was evaluated by two independent reviewers using the COnsensus-based Standards for the selection of health Measurement INstruments (COSMIN) checklist. Quality of the questionnaires was rated per measurement property, based on the number and quality of the included studies and the reported results.

**Results:**

Of 6286 unique hits, 21 studies met our criteria evaluating nine different questionnaires in multiple settings and languages. The methodological quality of the included studies was variable for the different measurement properties: 9/15 studies scored ‘good’ or ‘excellent’ on internal consistency, 2/5 on reliability, 0/1 on content validity, 10/10 on structural validity, 8/11 on hypothesis testing, 1/5 on cross-cultural validity, and 4/9 on criterion validity. For the CES-D, there was strong evidence for good internal consistency, structural validity, and construct validity; moderate evidence for good criterion validity; and limited evidence for good cross-cultural validity. The PHQ-9 and WHO-5 also performed well on several measurement properties. However, the evidence for structural validity of the PHQ-9 was inconclusive. The WHO-5 was less extensively researched and originally not developed to measure depression.

**Conclusion:**

Currently, the CES-D is best supported for measuring depressive symptoms in diabetes patients.

**Electronic supplementary material:**

The online version of this article (10.1007/s11136-018-1782-y) contains supplementary material, which is available to authorized users.

## Introduction

Diabetes is a common and serious chronic disease that is estimated to affect more than 350 million people worldwide [1]. Adult patients with diabetes type 1 or type 2 often have comorbid depression. Up to 20% of diabetes patients have major depressive disorder and up to 40% have clinically relevant depressive symptoms at one point in time according to the criteria of the Diagnostic and Statistical Manual of Mental Disorders-IV (DSM-IV) [2–4].

Comorbid depression in patients with diabetes is associated with poorer adherence to medical treatment and more difficulties complying with self-care instructions compared to patients with diabetes alone [5]. These patients also experience adverse health outcomes, such as poorer glycemic control [6], more diabetes complications [7], lower quality of life [8] and higher risk of morbidity and all-cause mortality. Furthermore, they use more healthcare resources resulting in higher healthcare costs [9].

Given the high prevalence of comorbid depression and associated adverse health outcomes, it is important to monitor depressive symptoms in diabetes patients on a regular basis, for example to evaluate changes during and after an intervention. Clinical guidelines recommend doing this with standardized questionnaires [10]. This way, depression treatment can be optimized and adjusted when necessary [11].

A wide variety of questionnaires is available to measure depressive symptoms. Questionnaires that are frequently used in diabetic populations are the Center of Epidemiological Studies-Depression Scale (CES-D) [12], the Hospital Anxiety and Depression Scale-Depression (HADS-D) [13], the Patient Health Questionnaire-9 (PHQ-9) [14] and the Beck Depression Inventory-II (BDI-II) [15]. However, these questionnaires generally assess symptoms of depression that may overlap with common symptoms of a medical illness such as diabetes (e.g., fatigue, changes in weight and appetite). Although many of these questionnaires have undergone extensive psychometric testing, an overview of their performance in this specific diabetes population is lacking [16]. This information is valuable because measurement properties may vary across populations. Also, a large number of questionnaires is available, while no recommendations are available which one to use to monitor depressive symptoms in diabetes patients. This makes it difficult to select the most suitable questionnaire for monitoring and evaluating depressive symptoms in diabetes patients.

Systematic, comparative evidence on the measurement properties of these questionnaires, used for evaluating depressive symptoms within patients on a continuous scale, is required by physicians and researchers.

Measurement properties are divided in three domains: reliability, validity, and responsiveness [17]. A reliable questionnaire performs its measurements precisely, without too much measurement error. A valid questionnaire has the ability to measure the intended construct (and not something else). A responsive questionnaire is sensitive to changes in the construct to be measured [17]. Next to these three domains, it is important that a measurement instrument is interpretable, meaning that the quantitative results of the questionnaire can be translated to clinically meaningful conclusions [17].

Roy et al., conducted a comprehensive review in 2012 in which they identified frequently used depression questionnaires used in diabetes patients. They conclude that the BDI, PHQ-9, CES-D and the HADS-D are most used. However, they did not systematically evaluate the measurement properties of the included questionnaires. It is therefore not known which questionnaire is most reliable and valid for measuring depressive symptoms in diabetes patients. Therefore, the aim of this study was to summarize the comprehensive research on the measurement properties (reliability, validity and responsiveness) of questionnaires used to evaluate depressive symptoms in adult patients with type 1 and type 2 diabetes. Knowledge generated from this study may help clinicians and researchers to make a better evidenced-based selection of questionnaires for the evaluation of depressive symptoms among diabetes patients.

## Methods

### Design

A systematic review of the literature was conducted according to the recommendations from the COnsensus-based Standards for the selection of health status Measurement INstruments (COSMIN) initiative (https://www.cosmin.nl). According to these recommendations, the literature was systematically searched; the quality of the included studies was assessed; the results of the studies were rated against predefined criteria; the results of multiple studies per measurement property were systematically synthesized, and levels of evidence were applied. A detailed description of the used methods is provided below.

### Data sources, search strategy, and study selection

We searched MEDLINE, EMBASE, and PSYCINFO from inception [i.e. with no specified beginning date up until (and including)] to October 2016. The investigators developed the search after consulting an information specialist (a university librarian). The search strategy consisted of search terms for depression and type 1 and type 2 diabetes. Different from the COSMIN recommendations, terms regarding type of instrument and measurement properties (reliability, validity and responsiveness) were not used, because we wanted to reduce the chance of missing any relevant articles. The used search terms are shown in S1 Appendix.

We included all studies published in any language on the measurement properties of self-report questionnaires measuring depressive symptoms (as defined as such by the authors of the paper) in type 1 or type 2 diabetes patients (i.e. at least 80% of the study population had diabetes). Studies were included in the review when the questionnaire under study was used to measure depressive symptoms, even if the questionnaire was not originally developed for this purpose. Only studies that reported measurement properties of these questionnaires, i.e. reliability, validity and/or responsiveness, were included in the review. Studies that only assessed the diagnostic accuracy of a questionnaire were not included, since these studies are concerned with the ability of a questionnaire to detect a target condition, while in this review, the focus is on the evaluative use of questionnaires to monitor the severity of depressive symptoms over time.

Three reviewers (SD, LZ, MA) independently assessed the titles and abstracts of the retrieved studies to identify relevant studies. The reviewers worked in pairs and discussed their selection. When both reviewers agreed a study was possibly relevant or when consensus was not reached, the full text article was retrieved and read by all three authors to determine whether in- and exclusion criteria were met. For the final inclusion of an article, after reading of the full text, consensus between all three reviewers (SD, LZ and MA) was needed. When consensus was not reached, a fourth reviewer decided (CT). The reference lists of the included articles were checked by two reviewers independently of each other (SD and LZ) and related citations of relevant articles found in MEDLINE were screened to identify additional relevant studies.

### Identification of studied measurement properties

Two reviewers (SD and LZ) independently identified for each study which measurement properties were reported. When no consensus was reached, a third reviewer discussed the interpretation of the reviewers with them and decided based on her leading expertise in the field of measurement properties (CT). Based on the COSMIN recommendations, three domains of measurement properties were distinguished: reliability, validity and responsiveness [17].

#### Reliability

A self-reported health questionnaire is considered reliable when it (a) is internally consistent, with all items (in a subscale) showing a high degree of interrelatedness (Cronbach’s *α* .70–.90); (b) has high reliability, which means that a high proportion of the variability in the measurement outcome is caused by real differences between or changes within patients; and (c) does not introduce a lot of measurement error (differences in the measurement outcome that cannot be attributed to differences in the construct to be measured [17]).

#### Validity

Validity of a questionnaire includes (a) content validity, or how well a questionnaire reflects the construct it is supposed to measure; (b) construct validity, or to which degree the measurement outcome reflects the dimensional structure of a questionnaire (structural validity), the degree to which the scores of a questionnaire are consistent with hypotheses based on theoretical knowledge of the construct to be measured (hypothesis testing) and the degree to which a translated questionnaire performs similarly to the original version (cross-cultural validity); and (c) criterion validity, or how well the outcome of a questionnaire reflects the outcome of a ‘gold standard’ to measure the same construct [17].

#### Responsiveness

A questionnaire is considered responsive when it is able to detect change in the construct to be measured.

Next to these three domains of measurement properties, it is important that the results of a questionnaire are interpretable [17].

### Assessment of the methodological quality of the included studies

After consensus was reached on which properties were assessed in the selected studies, the methodological quality of the assessment of each studied measurement property was rated for all studies using the COSMIN checklist [18]. This checklist consists of 9 boxes that correspond with the defined measurement properties. In each box, methodological standards are presented on how each measurement property should be assessed. The 9 boxes consist of 5 (content validity) through 18 (hypothesis testing) items. These items are scored in a standardized way on a 4-point scale (i.e. “poor”, “fair”, “good” or “excellent”) [19]. An overall score of the methodological quality for each box was determined by taking the lowest rating of any of the items in that box, since a low rating on any of the items signals a significant risk of bias. The quality assessment was independently done by three reviewers who worked in pairs (SD and LZ; SD and MA). These reviewers were trained by one of the developers of the COSMIN checklist (CT). A third reviewer (CT) decided when consensus on any item was not reached.

### Data extraction

Characteristics of the study design and questionnaires were extracted and summarized for all included studies. Study design characteristics included questionnaire used, sample size, mean age, gender distribution, proportion of diabetes patients in the sample, country and setting in which the study was performed and language version of the used questionnaire. The following questionnaire characteristics were extracted: construct aimed to be measured, target population, number of items, subscales of the questionnaire, score range of the items and total scores, usual cut-points for depression, administration time and recall period.

The results regarding the reported measurement properties were extracted by two reviewers (SD and LZ), independently. Results on the instrument quality were abstracted for every measurement property separately using a standardized data extraction form. We used common criteria to classify results as positive (good measurement quality of the questionnaire), inconclusive or negative (poor measurement quality of the questionnaire) [20]. The used criteria for quality of measurement properties can be viewed in S2 Appendix.

### Data synthesis

To rate the overall quality of the questionnaires, we combined the results on each measurement property with the ratings of methodological quality in each box, the number of studies in which the measurement property was investigated and the consistency of the results. In the data synthesis, only results of studies of excellent, good or fair methodological quality are considered, as recommended by the COSMIN initiative. An overall ‘level of evidence’ per measurement property was assigned to each individual questionnaire in accordance with previously performed systematic literature reviews [21, 22]. As a result of this process, measurement properties were rated as positive, inconclusive or negative, with strong, moderate, limited or unknown level of evidence. In Table 1, the criteria used in this rating system are further explained. Although questionnaires are often evaluated using different language versions and their measurement properties are not necessarily similar across countries, results were summarized for every questionnaire, regardless of language version because there were not enough data to study differences in measurement properties between language versions. The data synthesis was independently performed by two reviewers (SD and MT) and in case consensus was not reached, a third reviewer (CT) made a final decision taking the arguments of the other reviewers into account.

**Table 1:** Criteria for assigning a level of evidence rating

Level of evidence	Rating	Criteria
Strong	+++ or − − −	Consistent findings in multiple studies of good methodological quality, or in one study of excellent methodological quality
Moderate	++ or − −	Consistent findings in multiple studies of fair methodological quality, or in one study of good methodological quality
Limited	+ or −	Evidence from one study of fair methodological quality
Inconclusive	+/−	Inconclusive evidence
Unknown	?	Only studies of poor methodological quality

Criteria were based on previously performed systematic reviews [21, 22]

## Results

### Search strategy, inclusion of relevant studies and studied questionnaires

**Fig. 1:**
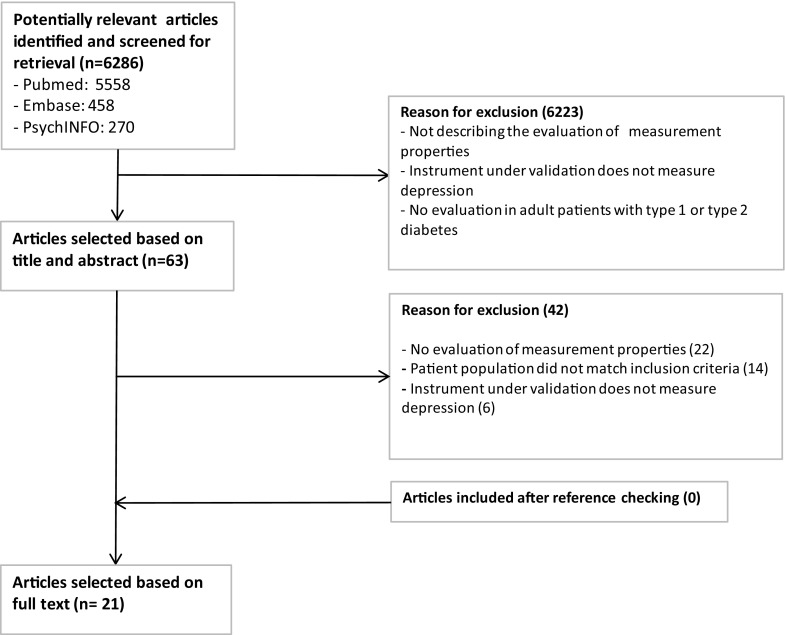
Selection of studies flowchart

The search strategy yielded 6286 unique articles, of which 63 were selected based on title and abstract. After reading the full text version, 21 were eligible for inclusion. Searching related citations in MEDLINE and reference lists of included papers yielded no additional articles. Thus, in total, 21 relevant studies were included in this review [23–43]. The inclusion process is described in more detail in Fig. 1.

Nine different questionnaires were evaluated: the CES-D [12], the Chinese version of the Clinically Useful Depression Outcome Scale (CUDOS) [44], the Depressive Cognition Scale (DCS) [45], the Depression in Diabetes Self-Rating Scale (DDSRS) [27], the Edinburgh Depression Scale [46], the HADS-D [13], the McSad [47], the PHQ-9 [14] and the 5-item World Health Organisation Well Being Index (WHO-5) [48]. Information regarding the selected articles and the depression questionnaires is presented in Tables 2 and 3.

**Table 2:** Characteristics of the included studies

Studies in alphabetic order	Instruments	Sample size	Mean age in years (SD)	Male (%)	DM1/DM2 (% of total sample)	Country in which study was performed	Setting	Language
Awata et al. [23]	WHO-5	129 65 (criterion validity)	54 (10)	55	16/84	Japan	University hospital	Japanese
Carter et al. [42]	CES-D	305	56.9 (11.1)	45	1/100	Canada	Rehabilitation institute	English
de Cock et al. [24]	EDS	1656	65/67 (10/10.6)^a^	50	0/100	The Netherlands	Primary care	Dutch
Hajos et al. [25]	WHO-5	933	53.4	49	41/59	The Netherlands	Hospital outpatient clinic	Dutch
Hsu et al. [26]	CUDOS	214	62.6 (13.2)	45	0/100	Taiwan	University hospital outpatient clinics	Chinese
Janssen et al. [43]	PHQ-9	793	62.4 (7.7)	67	0/100	The Netherlands	Community-based sample	Dutch
Kokoszka [27]	DDSRS	101	63 (11)	50	0/100	Poland	Medical University	Polish
Lamers et al. [28]	PHQ-9	365 (internal consistency, criterion validity) 226 (hypothesis testing) 27 (reliability)	71 (6.9)^b^	52^b^	0/100	The Netherlands	Primary care	Dutch
Lehman et al. [29]	CES-D	151	56 (10)	46	0/100	Turkey	University hospital outpatient clinics	Turkish
Lloyd et al. [30]	PHQ-9, WHO-5	24	55	50	0/100	UK (Bangladeshi and Pakistani)	Hospital outpatient clinic	Sylheti, Mirpuri
Papageorgiou et al. [31]	McSad	114	44 (14.1)	22	?/?^c^	The Netherlands	Members of a diabetes patient organisation	Dutch
Rankin et al. [32]	CES-D	30	range 46 thru 80	57	0/100	United States	Comprehensive health care centre	Chinese
Reddy et al. [33]	PHQ-9, HADS-D	462 (PHQ-9)/561 (HADS-D)	70	55	0/100	Australia	Primary care	English
Sousa et al. [34]	DCS	40	29.25 (10.23)	30	?/?^c^	Brazil	Convenience sample	Portuguese
Sousa et al. [35]	DCS	82	61.28 (11.37)	35	?/?^c^	Brazil	Primary care	Portuguese
Stahl et al. [37]	CES-D	522 (internal consistency) 291 (criterion validity)	55(13)	–	3.5/96.5	USA	Hospital diabetes centre	Chinese, Malay, Indian
Sultan and Fisher [36]	CES-D	502	53.6 (8.8)	54	0/100	USA	Community based sample	English, Spanisch
Zauszniewski et al. [38]	CES-D	80	82	30	0/100	USA	Hospital	English
Zauszniewski and Graham [39]	DCS	83	46	0	?/?^c^	USA	Hospital	English
Zhang et al. [40]	PHQ-9	586 (internal consistency) 40 (reliability) 99 (criterion validity)	55.1 (9.5)	59	0/100	China	Hospital outpatient clinic	Chinese
Zhang et al. [41]	CES-D	545 (internal consistency, structural validity) 40 (reliability) 97 (criterion validity)	54.6 (9.5)	59	0/100	China	Hospital outpatient clinic	Chinese

*CES-D* Centre for Epidemiological Studies Depression Scale, *CUDOS* Clinically Useful Depression Outcome Scale, *DCS* Depression Cognition Scale, *DDSRS* Depression in Diabetes Self-Rating Scale, *DM1* diabetes mellitus type 1, *DM2* diabetes mellitus type 2, *EDS* Edinburgh Depression Scale, *HADS-D* Hospital Anxiety and Depression Scale-depression, *PHQ-9* Patient Health Questionnaire-9, *SD* standard deviation, *UK* United Kingdom, *USA* United States of America, *WHO-5* World Health Organization-Five Well-Being Index

^a^Mean and standard deviation reported separately for male/female participants

^b^Characteristics of the total cohort in the study (not only diabetes patients) (*N* = 713)

^c^No details were reported on the number of type 1 and type 2 diabetes patients. However, the total sample consisted of 100% diabetes patients (either type 1 or type 2)

**Table 3:** Characteristics of the included questionnaires

Name	Construct aimed to be measured	Target population	# Items	Subscales	Score range (item level)	Score range (total)	Usual cut-points for depression	Administration time (min)	Recall period
CES-D	Level of depressive symptomatology	General population	20	NA	0–3	0–60	≥ 16	5–10	1 week
CUDOS	Depressive symptoms	General population	18	NA	0–4	0–72	0–10: no depression 11–20: minimal 21–30: mild 31–45: moderate > 45: severe	3	1 week
DCS	Depressive cognitions	Older adults	8	NA	0–5	0–40	≥ 7^a^	Not specified	NA
DDSRS	Depressive symptoms	Diabetes patients	6	NA	0–4	0–24	0–2 low severity^a^ 3–10 severe^a^ 11–24 high severity^a^	< 5	1 week
EDS	Depressive symptoms	Originally: women, post natal Later: several different patient groups	10	NA	0–3	0–30	0–8 not depressed 9–11 minor depression ≥ 12/13 major depression	A few minutes	1 week
HADS	Depression/anxiety	Hospital outpatients	14 (7 in every subscale)	Depression anxiety	0–3	0–42 (0–21 in every subscale)	8–10 on depression subscale	2–5	1 week
McSad	Major unipolar depression for valuation purposes	General population	6 (1 per subscale)	Emotion self-appraisal Cognition physiology Behavior Role function	1–4	Classification into 1 of 4096 descriptive profiles. In current study a total score of 0–24 is used	Not specified	A few minutes	1 week
PHQ-9	Symptoms of major depressive disorder	Primary care patients	9	NA	0–3	0–27	0–4: no depression 5–9: minimal 10–14: mild 15–19: moderate ≥ 20: severe	Within minutes	2 weeks
WHO-5	Emotional well-being (and later depression)	General population	5	NA	0–5	0–25	≤ 13	< 5	2 weeks

*CES-D* Centre for Epidemiological Studies Depression Scale, *CUDOS* Clinically Useful Depression Outcome Scale, *DCS* Depression Cognition Scale, *DDSRS* Depression in Diabetes Self-Rating Scale, *EDS* Edinburgh Depression Scale, *HADS-D* Hospital Anxiety and Depression Scale-Depression, *NA* not applicable, *PHQ-9* Patient Health Questionnaire-9, *WHO-5* World Health Organization-Five Well-Being Index

^a^No usual cut-off points specified, but cut-off points resulted from analyses in included studies

Sample sizes of the included studies varied widely, ranging from 24 [30] to 1656 [24]. The population in which the questionnaires were assessed differs greatly regarding age, languages and settings. For example, mean age of the participants ranged from 29 [34] to 82 [39]. The questionnaires were assessed in ten different languages (Japanese [23], Dutch [24, 25, 28, 31, 43], Chinese [26, 32, 37, 40, 41], Polish [27], Turkish [29], Sylheti [30], Mirpuri [30], English [33, 36–39, 42], Portuguese [34, 35] and Spanish [36]). The setting in which the questionnaires were researched differed between studies (for example, primary care, hospital outpatient clinics, university hospitals and patient support group organizations). Most samples only consisted of type 2 diabetes patients, but three studies also included type 1 diabetes patients [23, 25, 37]. Four studies did not specify the number of type 1 and type 2 diabetics [31, 34, 35, 39].

### Methodological quality

The methodological quality of the studies was variable ranging from ‘poor’ to ‘excellent’ (Table 4). Structural validity was rated as ‘good’ and ‘excellent’ for all studies. The most frequently assessed measurement properties were internal consistency (17 studies [23, 25–29, 32–41, 43]) and hypothesis testing (13 studies [23, 25–29, 31–33, 35, 36, 38, 39]). Only few studies examined reliability (5 studies) [26, 28, 33, 40, 41], cross-cultural validity (5 studies) [23, 26, 30, 32, 34] and content validity (1 study) [30]. There were no studies that examined measurement error, responsiveness or interpretability.

**Table 4:** Methodological quality of the included studies per measurement property

Publication	Used questionnaire	Internal consistency	Reliability	Content validity	Structural validity	Hypotheses testing	Cross-cultural validity	Criterion validity
Awata et al. [23]	WHO-5	Good			Good	Fair	Fair	Poor
Carter et al. [42]	CES-D				Good			
de Cock et al. [24]	EDS				Good			
Hajos et al. [25]	WHO-5	Excellent			Excellent	Good		Poor
Hsu et al. [26]	CUDOS	Good	Good		Good	Fair	Fair	Fair
Janssen et al. [43]	PHQ-9	Poor			Good			Good
Kokoszka [27]	DDSRS	Poor				Poor		Poor
Lamers et al. [28]	PHQ-9	Poor	Poor			Fair		Good
Lehman et al. [29]	CES-D	Good			Good	Good		
Lloyd et al. [30]	PHQ-9/WHO-5			Poor			Poor	
Papageorgiou et al. [31]	McSad					Excellent		
Rankin et al. [32]	CES-D	Poor				Poor	Fair	
Reddy et al. [33]	PHQ-9/HADS-D		Excellent		Excellent	Good		
Sousa et al. [34]	DCS	Fair					Good	
Sousa et al. [35]	DCS	Good			Good	Good		
Stahl et al. [37]	CES-D	Poor						Fair
Sultan and Fisher [36]	CES-D	Good			Good	Good		
Zauszniewski et al. [38]	CES-D	Good				Good		
Zauszniewski and Graham [39]	DCS	Good				Good		
Zhang et al. [40]	PHQ-9	Fair	Poor					Good
Zhang et al. [41]	CES-D	Poor	Poor		Good			Good

*CES-D* Centre for Epidemiological Studies Depression Scale, *CUDOS* Clinically Useful Depression Outcome Scale, *DCS* Depression Cognition Scale, *DDSRS* Depression in Diabetes Self-Rating Scale, *EDS* Edinburgh Depression Scale, *HADS-D* Hospital Anxiety and Depression Scale-Depression, *PHQ-9* Patient Health Questionnaire-9, *WHO-5* World Health Organization-Five Well-Being Index

Two out of 15 studies scored ‘excellent’ [25, 33] and seven studies scored ‘good’ [23, 26, 29, 35, 36, 38, 39] on internal consistency, Lower quality ratings were mostly caused by not assessing or describing the dimensionality of a questionnaire and not assessing internal consistency for every subscale of a questionnaire separately [27, 28, 32, 37, 40, 41, 43], or having a small or not representative study population [32, 34].

Five studies assessed the reliability of the investigated questionnaire, of which one study was rated ‘excellent’ [33] and one study was rated ‘good’ [26]; the other three studies were of poor methodological quality [28, 40, 41] due to flaws in the study design or statistical methods used.

One study [30] reported content validity and was rated ‘poor’ due to methodological flaws in the design of the study. Of the ten studies reporting structural validity [23–26, 29, 33, 35, 36, 41], two were rated ‘excellent’ [25, 33] and the other eight studies were rated ‘good’. The difference between a ‘good’ and ‘excellent’ rating was caused by differences in reporting on missing values and drop-out in the study, or differences in sample size, with excellent studies having a larger sample.

One out of 11 studies reporting hypothesis testing was rated ‘excellent’ [31]. Seven studies were rated ‘good’ [25, 29, 33, 35, 36, 38, 39]. The main reasons for a lower quality score were small sample size [28, 32] or not sufficiently specifying prior hypotheses [23, 26, 27].

Of the five studies assessing cross-cultural validity, one study was rated ‘good’ [34]. Lower quality scores were mainly caused by flaws in the translation process, not testing the translation in patients with diabetes before using the questionnaire in this population [23, 26, 32] and small sample size [30].

Nine studies assessed criterion validity; four of these studies scored ‘good’ [28, 40, 41, 43]. The main reasons for a poor or fair rating were not using an accurate reference standard for measuring depression [25, 27], flaws in the study design [23, 27] or using a case control design without correction [23], thereby inflating estimates of criterion validity [49]. None of the studies were rated excellent because no gold standard exists to measure depression.

### Measurement properties of questionnaires measuring depressive symptoms

Table 5 summarizes all results on measurement properties for each questionnaire. The final judgment on the level of evidence for the quality of the questionnaires per measurement property is presented in Table 6. Since none of the studies assessed measurement error, responsiveness or interpretability, these properties are not included in the tables.

**Table 5:**
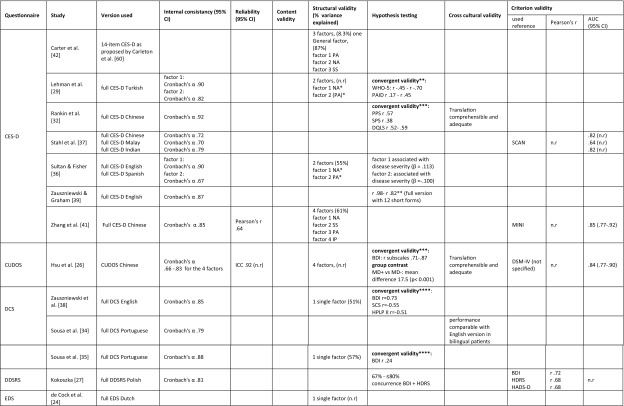

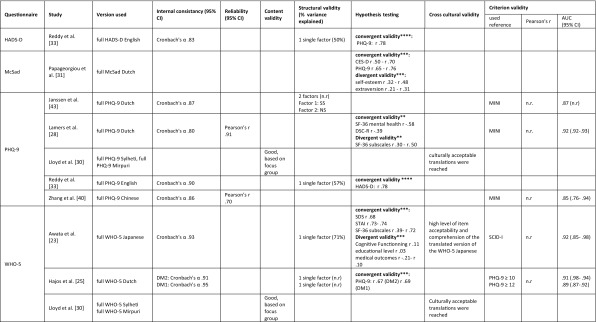
Results of all assessments of measurement properties, organized by questionnaire

*95% CI* 95% confidence interval, *AUC* area under the curve, *BDI* Beck Depression Inventory, *CES-D* Centre for Epidemiological Studies Depression Scale, *cog*. cognitive, *CSDD* Scale for the Diagnosis of Depression, *CUDOS* Clinically Useful Depression Outcome Scale, *DCS* Depression Cognition Scale, *DDSRS* Depression in Diabetes Self-Rating Scale, *DM1* diabetes mellitus type 1, *DM2* diabetes mellitus type 2, *DQLS* Diabetes Quality of life Scale, *DSC-R* diabetes symptom checklist-revised, *DSM-IV* diagnostic and statistical manual of mental disorders, fourth edition, *EDS* Edinburgh Depression Scale, *HDRS* Hamilton Depression Rating Scale, *HADS-D* Hospital Anxiety and Depression Scale-Depression, *HPLP-2* health promoting lifestyle profile-II, *ICC* intra class correlation, *IP* interpersonal problems, *n.r*. not reported, *MD* major depression, *MINI* Mini International Neuropsychiatric Interview, *MSA* Mokken Scale Analysis, *NA* negative affect, *n.r*. not reported, *NS* non-somatic symptoms, *PA* positive affect, *PAID* problem areas in diabetes, *PCA* principal component analysis, *PHQ-9* Patient Health Questionnaire, *PPS* Pscychological Problems Scale, *SCAD* silverstone concise assessment for Depression, *SCAN* schedules for clinical assessment in neuropsychiatry, *SCID* structured clinical interview for DSM, *SCS* self-control schedule, *SDS* Zung’s Self-Rating Depression Scale, *SF-36* medical outcomes study 36-item short form health survey, *SPS* Social Problems Scale, *SS* somatic symptoms, *STAI* State-Trait Anxiety Inventory, *WHO-5* World Health Organization-Five Well-Being Index

*Eigenvalue factor 1 (negative affect): 7.345, factor 2 (positive affect) 2.249

**Pearson’s correlation coefficient

***Spearman’s correlation coefficient

****Type of correlation coefficient not reported

**Table 6:** Levels of evidence for the quality of the questionnaires

	Internal consistency	Reliability	Content validity	Structural validity	Hypothesis testing	Cross-cultural validity	Criterion validity
CES-D	+++	NA	NA	+++	+++	+	++
CUDOS	++	++	NA	− −	+	+	+
DCS	+++	NA	NA	+++	+/−	++	NA
DDSRS	?	NA	NA	NA	?	NA	?
EDS	NA	NA	NA	++	NA	NA	NA
HADS	+++	NA	NA	+++	++	NA	NA
McSad	NA	NA	NA	NA	+++	NA	NA
PHQ**−**9	+++	?	?	+/−	++	?	+++
WHO-5	+++	?	?	++	++	+	?

*+++* strong positive evidence; *++* moderate positive evidence; *+* limited positive evidence; − − −strong negative evidence; *− −* moderate negative evidence; *+/−* inconclusive; *?* unknown, due to poor methodological quality; *NA* no information available

#### CES-D

The CES-D was assessed in six different languages in six studies [29, 32, 36, 37, 39, 41]. For internal consistency, structural validity, hypothesis testing, cross-cultural validity and criterion validity, there is predominantly strong to moderate evidence for good performance of the CES-D. Although not all studies assessing structural validity found the same factor structure, the two dominant factors (positive affect and negative affect) were found in every study [29, 36, 41, 42]. The additional factors found by Zhang et al. [41] and Carter et al. [42] all correlate highly with the negative affect factor. Therefore, we consider the evidence on structural validity consistent. One study evaluated reliability, but was of poor methodological quality [41]. Therefore, it was not possible to draw conclusions about the reliability of the CES-D.

#### CUDOS

The measurement properties of the CUDOS-Chinese were assessed in one study [28]. Results for internal consistency, reliability, structural validity, hypothesis testing and criterion validity were available. For internal consistency, inconsistent findings on four subscales resulted in inconclusive evidence. In confirmatory factor analysis, four subscales were found, and as far as we know current literature does not support the existence of four subscales in the depression construct. Therefore, structural validity was considered poor, with moderate evidence supporting this finding. Reliability, construct validity (hypothesis testing) and criterion validity were good for the CUDOS-Chinese, with moderate to limited evidence for these findings.

#### DCS

The DCS was evaluated in three different studies, using two different languages (English and Portuguese) [34, 35, 38]. There was strong evidence for good internal consistency and structural validity. One study showed moderately strong evidence of good cross-cultural validity of the Portuguese translation of the DCS [34]. Hypothesis testing resulted in inconclusive findings. Reliability, measurement error, content validity, responsiveness and interpretability were not assessed.

#### EDS

Only one study [24] assessed a Dutch version of the EDS. Within this study only structural validity was assessed. Since this was done with good methodological quality and the analysis yielded one single, theoretically explicable factor, evidence regarding structural validity was considered moderate for good structural validity.

#### HADS-D

One single study [33] assessed measurement properties of the HADS-D. There was strong evidence for good internal consistency and structural validity and moderate evidence for good construct validity (hypothesis testing).

#### McSad

The construct validity of the Dutch McSad was evaluated in one study using hypothesis testing [31]. The methodological quality of this assessment was rated excellent, resulting in confirmation of all pre-set hypothesis. The level of evidence was therefore rated ‘strong’ for good construct validity.

#### PHQ-9

Measurement properties of the Patient Health Questionnaire (PHQ) were assessed in five different studies in five different languages [28, 30, 33, 40, 43]. Reliability, content validity, cross-cultural validity, internal consistency, structural validity and criterion validity were assessed. However, assessments of reliability, content validity and cross-cultural validity were not included in the data synthesis, since these were of poor methodological quality. There was strong evidence of good internal consistency and criterion validity. Construct validity (hypothesis testing) was rated ‘good’ with a moderate level of evidence. The evidence for structural validity was inconclusive, since two studies of at least good quality found different factor structures [33, 43].

#### WHO-5

Measurement properties of the WHO-5 were assessed by three different studies in four different languages [23, 25, 30]. Reliability, measurement error, responsiveness and interpretability were not assessed and the assessments of content validity and criterion validity were of poor methodological quality. Internal consistency was good, with strong level of evidence. Evidence for good structural validity and construct validity (hypothesis testing) was moderate. There was limited evidence for good cross-cultural validity of the WHO-5 [23].

## Discussion

We identified 21 studies evaluating the measurement properties of nine different questionnaires for measuring depressive symptoms in diabetes patients. Overall, the CES-D performed best, with strong evidence for a positive internal consistency, structural validity, and construct validity, moderate evidence for a positive criterion validity and limited evidence for positive cross-cultural validity. Insufficient information was available on content validity and reliability.

The use of the WHO-5 was supported by strong evidence for a positive internal consistency and moderate evidence for a positive structural validity and construct validity. However, the WHO-5 is originally developed as a questionnaire to measure the level of emotional well-being and not to assess depressive symptoms. Yet, caution should be applied when choosing the WHO-5 to specifically measure depressive symptoms. The PHQ-9 is frequently studied amongst patients with diabetes. We found strong evidence for a positive internal consistency and positive criterion validity and moderate evidence for positive construct validity. However, since the evidence for its structural validity is inconclusive, caution should be applied when the PHQ-9 is used for evaluative purposes. For all other questionnaires, evidence is too limited to draw any definitive conclusions regarding their measurement properties. Therefore, based on the current evidence, we recommend using the CES-D for evaluating depressive symptoms in patients with diabetes. However, for none of the questionnaires complete information is available on all measurement properties when used in a population of adults with diabetes. One important shortcoming is lack of evidence on the content validity of the questionnaires, including the CES-D, in diabetes patients. Content validity is often considered the most important measurement property because it can affect all other measurement properties. Therefore, we recommend further literature review on the content validity of these questionnaires in other populations, as well as qualitative studies with patients and professionals on the relevance, comprehensiveness, and comprehensibility of these questionnaires in diabetes patients. Furthermore, measurement error, responsiveness and interpretability were not assessed for any of the questionnaires. This is important since shortcomings in any of the measurement properties pose a considerable threat to the ability of a questionnaire to measure depression in diabetes patients [50].

Our systematic review adds to the current literature by providing a structured and comprehensive overview of the measurement properties of depression questionnaires used in diabetes patients and the methodological quality of the studies assessing them. Also, this review provides recommendations on their use. By describing which questionnaires are—at this point in time—best supported by the evidence, this review is of use when choosing a questionnaire to monitor depression in daily practice. Previously, Roy et al. conducted a comprehensive review of depression screening questionnaires and their operating characteristics in diabetes populations [16]. In their review, 23 relevant studies were identified. There is only limited overlap in studies (*n* = 5) between the study of Roy et al., and our review. This is mainly because we included studies that assessed measurement properties of questionnaires used to evaluate depressive symptoms (for evaluative purposes), and we excluded studies assessing diagnostic test accuracy of questionnaires used for screening or detecting a depressive disorder. Roy et al. concluded that there is lack of evidence on the reliability and validity of depression questionnaires used for patients with diabetes to provide recommendations. In our more recent and up-to-date review, more evidence was available to provide recommendations for measuring change in depressive symptoms.

Other reviews assessing the use of depression questionnaires in patients with chronic medical illnesses (for example, in patients with cancer [51] and Parkinson’s disease [52]) provided comparable recommendations, suggesting that our findings are robust. However, we are aware that we need to be cautious in recommending the use of the CES-D because of the lack of evidence on some important measurement properties, like reliability and responsiveness.

A new development in measuring and monitoring patient-reported health is the use of item banks based on Item Response Theory (IRT), such as those from the Patient Reported Outcomes Measurement Information System (PROMIS) (http://www.healthmeasures.net/promis). IRT-based item banks enable Computer Adaptive Testing (CAT), in which, after a starting question, the computer selects subsequent questions based on the answers to previous questions. With CAT patients need to complete on average only 5–9 questions to get a reliable score, which makes this method a highly efficient and patient-friendly way of measuring. The PROMIS Depression instruments seem to be valid and reliable for measuring depressive symptoms [53, 54]. Recent studies indicate that the PROMIS Depression CAT can be more easily used in clinical practice than the CES-D and PHQ-9 since it can be adapted to the needs in a specific care setting, while it results in comparable scores [55–57]. The PROMIS methodology is promising for use in patients with a chronic physical illness, minimizing the impact of somatic symptoms on depression scores while retaining enough uniformity to compare between patient populations and other depression measures [57]. Therefore, in time, PROMIS might replace the traditional depression questionnaires.

This study is the first to systematically summarize the evidence on the measurement properties of questionnaires measuring depressive symptoms in patients with diabetes. A strength of this study is the use of the standardized COSMIN methodology for critical appraisal of the methodological quality of these studies, the quality of the questionnaires and the level of evidence. Another strength of this review is its inclusive search strategy, thereby limiting chances of missing important studies.

The following two limitations apply. Firstly, the identified depression questionnaires were assessed in a large variety of languages and settings, but whether the results on the individual questionnaires discussed in this review can be validly generalized across language versions is not clear. Only few identified studies performed a cross-cultural validation of translated questionnaires in a diabetes population. Questionnaires may perform differently across different languages and cultures [21].

Secondly, to provide a comprehensive overview of measurement properties of depression questionnaires in diabetes patients, we deliberately excluded studies that only assessed diagnostic accuracy. Although this distinction is based on a theoretically sound concept and rests on differences in the use and purpose of a questionnaire (monitoring vs. diagnosing), in the various studies, this distinction was often not clearly made. This resulted in some difficulties deciding whether or not a study should be included in the review.

Further studies are needed on the measurement properties of depression questionnaires in diabetes patients. The finding that internal consistency, hypothesis testing and structural validity are the most evaluated properties is in line with other literature [22, 58, 59]. However, not all measurement properties (measurement error, responsiveness and interpretability) are extensively evaluated and further research is needed to provide definitive recommendations.

In summary, this systematic review constitutes an important knowledge base for health care providers and researchers by providing a comprehensive overview of questionnaires measuring depressive symptoms in diabetes patients. The CES-D has the strongest evidence for good measurement properties for measuring depressive symptoms in patients with diabetes.

## Electronic supplementary material

Below is the link to the electronic supplementary material.

Supplementary material 1 (DOCX 493 KB)

Supplementary material 2 (DOCX 484 KB)
