# Supplementary material for: Measurement properties of depression questionnaires in patients with diabetes: a systematic review
Source: Qual Life Res. 2018 Feb 2;27(6):1415–30. doi: 10.1007/s11136-018-1782-y (PMC5951879; doi:10.1007/s11136-018-1782-y)
Supplement: Supplementary file 1 — Supplementary material 1 (DOCX 493 KB) [file 11136_2018_1782_MOESM1_ESM.docx]

| \|  \|  \|  \|  \|  \| \| --- \| --- \| --- \| --- \| --- \| \|  \|  \|  \|  \|  \| \|  \|  \|  \|  \|  \| \|  \|  \|  \|  \|  \| |  |  |  |  |  |  |  |  |  |  |  |
| --- | --- | --- | --- | --- | --- | --- | --- | --- | --- | --- | --- | --- | --- | --- | --- | --- | --- | --- | --- | --- | --- | --- | --- | --- | --- | --- | --- | --- | --- | --- | --- |

**S1 Appendix. Data sources and search strategy**

| **Database** | **Date of search** | **Search terms** | **Selection options** | **Number of hits** | **Number of unique hits** |
| --- | --- | --- | --- | --- | --- |
| MEDLINE | Beginning of literature - October 26^th^ 2016 | *(1) construct*  #1: (Depressive disorder[mh] OR depression[mh] OR (depress*[tiab] NOT medline[sb]))  *(2) population*  #2: (Diabet*[tiab])  (#1 AND #2) | None used | 5719 | 5558 |
| EMBASE | Beginning of literature - October 26^th^ 2016 | #1: (Depression)  #2: (diabetes mellitus)  (#1 AND #2) | **Options:**  - Mapped to preferred term  - limit terms to index in article as ‘major focus’  - Explode  **Sources**:  - Embase  **Quick limits:**  - Humans  - With abstract  **All years**  **Pub. Types**  - Articles  - Articles in press  - Review | 1289 | 458* |
| PsychINFO | Beginning of literature - October 26^th^ 2016 | Depress* (title) AND diabetes (title) | None used | 526 | 270** |
| Total combined | Beginning of literature - October 26^th^ 2016 | Not applicable | Not applicable | 7534 | 6286 |

* hits that were already in the MEDLINE search were removed manually

**hits that were already in the MEDLINE or EMBASE search were removed manually
