# Supplementary material for: Measurement properties of depression questionnaires in patients with diabetes: a systematic review
Source: Qual Life Res. 2018 Feb 2;27(6):1415–30. doi: 10.1007/s11136-018-1782-y (PMC5951879; doi:10.1007/s11136-018-1782-y)
Supplement: Supplementary file 2 — Supplementary material 2 (DOCX 484 KB) [file 11136_2018_1782_MOESM2_ESM.docx]

**S2 Appendix. Used criteria for good quality of the measurement properties that are assessed in this review***

| Measurement property | Criteria for good quality |
| --- | --- |
| Internal consistency | *Positive rating:* Cronbach’s α is between .70 and .95 AND calculated for each unidimensional (sub)scale  *Indeterminate rating:* Cronbach’s α not calculated for each unidimensional (sub)scale OR dimensionality of the instrument is unknown  *Negative rating:* Cronbach’s α < .70 or > .95 in more than 25% of all unidimensional (sub)scales |
| Reliability | *Positive rating:* Intra Class Correlation (ICC) ≥.70 OR weighted Kappa ≥ .70 OR Pearson’s r ≥ .80  *Indeterminate rating:* No ICC OR weighted Kappa OR Pearson’s r determined  *Negative rating:* ICC <.70 OR weighted Kappa < .70 OR Pearson’s r <.80 |
| Content validity | *Positive rating:* Clear description of measurement aim, target population and construct to be measured AND target population and experts rate all items as relevant  *Indeterminate rating:* No clear description of measurement aim, target population and construct to be measured OR no target population or experts involved  *Negative rating* Target population OR experts rate any items as irrelevant or incomprehensible |
| Structural validity | *Positive rating:* Factors are theoretically plausible AND explained variance of the factor solution ≥ 50%  *Indeterminate rating:* Factors are theoretically plausible BUT explained variance of the factor solution is not reported  *Negative rating:* Factors are theoretically not plausible OR explained variance of the factor solution < 50% |
| Hypothesis testing | *Positive rating:* ≥ 75% of all predefined hypothesis are confirmed  *Indeterminate rating:* No hypothesis are defined  *Negative rating:* <75% of all predefined hypothesis are confirmed |
| Cross cultural validity | *Positive rating:* No important differences in performance compared to the original version  *Indeterminate rating:* Differences from original version not reported  *Negative rating:* Important differences compared to the original version. For example: a different factor structure is found. |
| Criterion validity | *Positive rating:* Area Under the Curve (AUC) ≥ .70 OR correlation with reference ≥ .70  *Indeterminate rating:* AUC OR Pearson’s r not determined  *Negative rating:* AUC <.70 OR correlation with reference < .70 |

*Based on previously published criteria [20] and systematic reviews [21, 22]
